# Identification of Hypoxia-Related Molecular Classification and Associated Gene Signature in Oral Squamous Cell Carcinoma

**DOI:** 10.3389/fonc.2021.709865

**Published:** 2021-11-23

**Authors:** Chen Li, Xin Chen, Xiaolin Ren, Jia-lin Chen, Hao Chen, Jing-jia Yu, Qiu-chi Ran, Shuang Kang, Xi-meng Chen, Zhen-jin Zhao

**Affiliations:** ^1^ Department of Orthodontics, The First Clinic of Stomatological Hospital of China Medical University, Shenyang, China; ^2^ Department of Neurosurgery, The First Hospital of China Medical University, Shenyang, China; ^3^ Department of Neurosurgery, Shenyang Red Cross Hospital, Shenyang, China

**Keywords:** oral squamous cell carcinoma (OSCC), hypoxia, subtyping, immune microenvironment, immunotherapy

## Abstract

The high heterogeneity of oral squamous cell carcinoma (OSCC) is the main obstacle for individualized treatment. Recognizing the characteristics of different subtypes and investigating the promising strategies for each subclass are of great significance in precise treatment. In this study, we systematically evaluated hypoxia-mediated patterns together with immune characteristics of 309 OSCC patients in the TCGA training set and 97 patients in the GSE41613 testing set. We further identified two different hypoxia subtypes with distinct immune microenvironment traits and provided treatment programs for the two subclasses. In order to assess hypoxia level individually, we finally constructed a hypoxia-related risk score, which could predict the clinical outcome and immunotherapy response of OSCC patients. In summary, the recognition of different hypoxia patterns and the establishment of hypoxia-related risk score might enhance our understanding of the tumor microenvironment of OSCC and provide more personalized treatment strategies in the future.

## Introduction

Oral squamous cell carcinoma (OSCC) is one of the most common malignant tumors of head and neck squamous cell carcinoma (HNSC), accounting for 90% of neoplasms of the head and neck (1). Despite the development of surgery, radiotherapy, and chemotherapy, the prognosis of OSCC is still unsatisfactory with an average 5-year survival probability ranging from 45% to 50% due to the high incidence of recurrence and metastasis (2–4). Recently, more and more studies have concentrated on the generation of genomic signatures for risk stratification and further survival prediction in OSCC patients (5–7). However, most prognostic signatures were deficient in clinical transformation and few of them were applied to routine practice. As a heterogeneous disease, it is of great necessity to precisely understand the molecular properties of OSCC in order to achieve individualized treatment under different subtypes.

Hypoxia is one of the critical hallmarks of cancer, which is associated with tumor malignancy and angiogenesis together with therapeutic resistance (8, 9). Currently, the significant role of hypoxia in driving tumor immunosuppression and immune escape has caused widespread concern. Evidence has revealed that T cells as well as natural killer (NK) cells under a hypoxia microenvironment always behave in an exhausted state, leading to their dysfunction in killing tumor cells (10). What is more, the hypoxia status can also promote some inhibitory immune cells like regulatory T cells (Tregs) and M2 macrophage infiltration together with the secretion of suppressive molecules like VEGFA, causing the formation of an immunosuppressive microenvironment (11–13). Even though hypoxia-related subclasses have been explored in many cancer types, the features of different subtypes and their clinical benefit in OSCC are still unknown. Therefore, investigating the distinct subtypes based on hypoxia status during tumorigenesis and development might provide new insights into the treatment and prognostic detection of OSCC.

Recently, immune checkpoint blockade (ICB) therapy has been reported to improve overall survival (OS) in distinct cancer types (14–20). Nevertheless, the proportion of benefited patients still remains low. Growing evidence has revealed a tight association between hypoxia and tumor immunotherapy across multiple tumor types (21). However, the effect of hypoxia on the immune microenvironment as well as the efficacy of immunotherapy in OSCC remains less known.

In the present study, a consensus clustering based on hypoxia genes was conducted and validated in two OSCC cohorts, characterizing two different hypoxia states of OSCC samples for the first time. Moreover, the prognostic features, hypoxia traits, gene mutation alterations, immune infiltration, and the promising treatment strategy for each subtype were analyzed and investigated. For clinical practice, we further constructed a hypoxia prognostic risk score model which could further predict the OS and ICB therapy response for OSCC patients. These findings suggested an indispensable role of hypoxia states in directing therapeutic plans for OSCC.

## Material and Methods

### Data Collection and Processing

The Cancer Genome Atlas (TCGA) mRNA sequence data [htseq-FPKM in log2(*x* + 1) transformed] together with clinical information of OSCC were obtained from the UCSC Xena browser (GDC hub: https://gdc.xenahubs.net). For validation, microarray profiles of GSE41613 containing clinical annotations were extracted by GEOquery R package. The mentioned clinical traits are demonstrated in **Table 1**. The batch effects normalized mRNA data of pancancer with clinical information were downloaded from UCSC Xena browser. The hypoxia gene set containing 200 classical hypoxia-associated genes was obtained from gene set enrichment analysis (GSEA) (http://www.gsea-msigdb.org/). Expression data of OSCC cell lines [TPM in log2(*x* + 1) transformed) were downloaded from the Broad Institute Cancer Cell Line Encyclopedia (CCLE) project (https://portals.broadinstitute.org/ccle/) (22). Drug sensitivity data (area under the curve—AUC) of OSCC cells from the Cancer Therapeutics Response Portal (CTRP v.2.0) and PRISM Repurposing dataset (19Q4) were acquired from the dependency map (DepMap) portal (https://depmap.org/portal/). The ICB treatment cohort GSE91061 (23) was downloaded from the GEO database [FPKM in log2(*x* + 1)] transformed and used for subsequent validation. The CheckMate 009 (CM-009), CheckMate 010 (CM-010), and CheckMate 025 (CM-025) (24) were combined together to investigate the significance of our risk score [FPKM in log2(*x* + 1)]. We also downloaded RNA-seq (count values) data of IMvigor210 cohort (25) with clinical information by the “IMvigor210CoreBiologies” R package and transformed it into FPKM values. The log2(FPKM + 1) was calculated on expression data for further comparison.

**Table 1:** Clinical and molecular information included in the study.

Cohort	TCGA-RNA-seq, OSCC (*n* = 309)	GSE41613, OSCC (*n* = 97)
Database	TCGA	GSE41613
Age (years)	61.82 ± 13.06	
Gender		
Male	209	66
Female	100	31
Overall survival (months)	29.72 ± 29.44	44.13 ± 26.52
Angiolymphatic invasion		
Yes	71	
No	159	
Unavailable	79	
Perineural invasion		
Yes	133	
No	109	
Unavailable	67	

### Consensus Clustering Analysis

Unsupervised clustering was applied to recognize different hypoxia patterns and classify OSCC patients for further analysis. A consensus hierarchical clustering algorithm based on the expression of 34 prognostic hypoxia genes was conducted by the “ConsensuClusterPlus” R package with Euclidean distance and Ward.D2’s linkage (number of bootstraps=50, item subsampling proportion = 0.8, feature subsampling proportion = 0.8).

### Survival Analysis

Univariate Cox regression analysis was conducted to identify prognostic hypoxia genes and clinical events. Multivariate Cox regression analysis was performed to recognize independent prognostic factors. The Kaplan–Meier survival curve was applied to analyze the prognostic significance between distinct groups.

### Single-Sample Gene Set Enrichment Analysis

The hypoxia-associated gene sets were downloaded from GSEA. The single-sample gene set enrichment analysis (ssGSEA) algorithm in “GSVA” R package was conducted to calculate the hypoxia score of each OSCC patient.

### Mutation Analysis

The MAF file of OSCC containing the detailed mutation information of the training set was downloaded from TCGA (https://portal.gdc.cancer.gov/) and further processed. The “maftool” R package was performed to analyze gene mutant features between two OSCC subclasses.

### Function Enrichment Analysis

The “Limma” R package was applied to identify differential genes between two clusters with a standard of |log FC| >1.2 and adjusted *P*-value <0.05. Further gene ontology (GO) function enrichment of selected genes was performed by ClueGO in Cytoscape.

### Tumor Microenvironment Analysis

The immune score and the tumor purity were calculated by the ESTIMATE algorithm (26). The CIBERSORT algorithm was applied to evaluate the *LM22* gene signatures in OSCC subtypes (27). What is more, the Epic algorithm was also used to calculate the contents of immune cell infiltration in the microenvironment (28).

### Screening Potential Agents of Cluster 2

k-Nearest neighbor (k-NN) imputation was performed to impute the missing AUC values of the CTRP and PRISM datasets. Before imputation, drugs with more than 20% of missing data were excluded. Furthermore, the “pRRophetic” R package was performed to measure the AUC values of samples by ridge regression.

### Development and Validation of Predictive Risk Score

Considering the difference of each platform, before developing or validating the risk score, we conducted z-scale of the mRNA data in each platform (TCGA, GSE41613, GSE91061, CM cohorts, and IMvigor210). Then, the “glmnet” R package was performed to filter the prognosis-related hypoxia genes by LASSO Cox regression analysis with a 10-fold cross-validation. After identifying the significant genes, their regression coefficients (*β*) were estimated by multivariate Cox regression *via* LASSO, and we calculated the risk score of each OSCC patient by the formula as follows:


$$\text{Risk score}=\Sigma_{i}Coefficient(mRNAi)\times Expression(mRNAi)$$

### Establishment of a Nomogram

Univariate Cox and multivariate Cox regression analyses of some clinical traits were first performed and finally determined a sum of four independent prognostic factors for further establishment. Afterward, a nomogram with the four factors was developed for predicting 1- and 3-year OS of OSCC patients. The calibration plot was performed to estimate the accuracy and consistency of the prognostic models. Survival net benefits of each variable were estimated with decision curve analysis (DCA) by “stdca.R.”

### Other Bioinformatics Analysis

Principal components analysis (PCA) was applied to verify the hypoxia patterns of different subtypes. Potential ICB response was predicted by the tumor immune dysfunction and exclusion (TIDE) algorithm (29). The “upsetR” R package was used to visualize the intersections between promising agents in different subtypes.

### Statistical Analysis

R 4.0.2 (https://www.r-project.org/) was mainly used for statistical analysis. Student’s *t*-test or one-way analysis of variance was used to analyze differences between groups in variables with a normal distribution. Categorical variables between two groups were compared using chi-square test. A two sided *P*-value <0.05 was considered statistically significant.

## Results

### Identification of Two Hypoxia-Associated Clusters in OSCC

**Figure 1:**
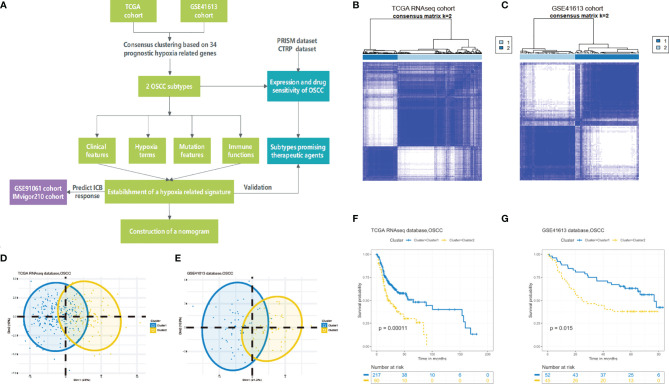
Identification of hypoxia-related clusters in oral squamous cell carcinoma (OSCC). **(A)** Overview of the analysis procedures. **(B, C)** Unsupervised clustering of OSCC patients based on the hypoxia-associated genes generated two clusters in the TCGA and GSE41613 cohorts. **(D, E)** Principal component analysis based on hypoxia genes distinguished two identified subtypes in different cohorts. **(F, G)** Kaplan–Meier survival analysis of overall survival between the two clusters in the two cohorts.

As depicted in **Figure 1A**, a brief flowchart was demonstrated to introduce our study. Considering the critical role of hypoxia condition in the tumor microenvironment, we summarized a sum of 188 classical hypoxia-stimulated genes available from GSEA and estimated their prognostic value for further classification (**Table S1**). Univariate Cox proportional hazards model was conducted and finally filtered 34 genes with significant risks on survival of patients in the training set (**Figures S1A, B**). Hence, based on the expression similarity of the 34 hypoxia-related gene signature, the consensus clustering method was used to cluster the samples. We selected *k* = 2 as the optimal number of clusters, which could divide all samples into two groups with less correlation between groups in the training and testing cohorts (**Figures 1B, C**). Then, PCA was conducted to compare the transcriptional profile between these two clusters in the two cohorts, suggesting a significant distinction between these two subgroups (**Figures 1D, E**). In order to evaluate the clinical relevance of this clustering, the survival analysis between the two subclasses was conducted. In these two sets, cluster 2 was consistently associated with worse prognosis, highlighting the potential clinical utility of this hypoxia-associated subtyping (**Figures 1F, G**).

### Distinct Hypoxia Conditions Between the Two OSCC Clusters

**Figure 2:**
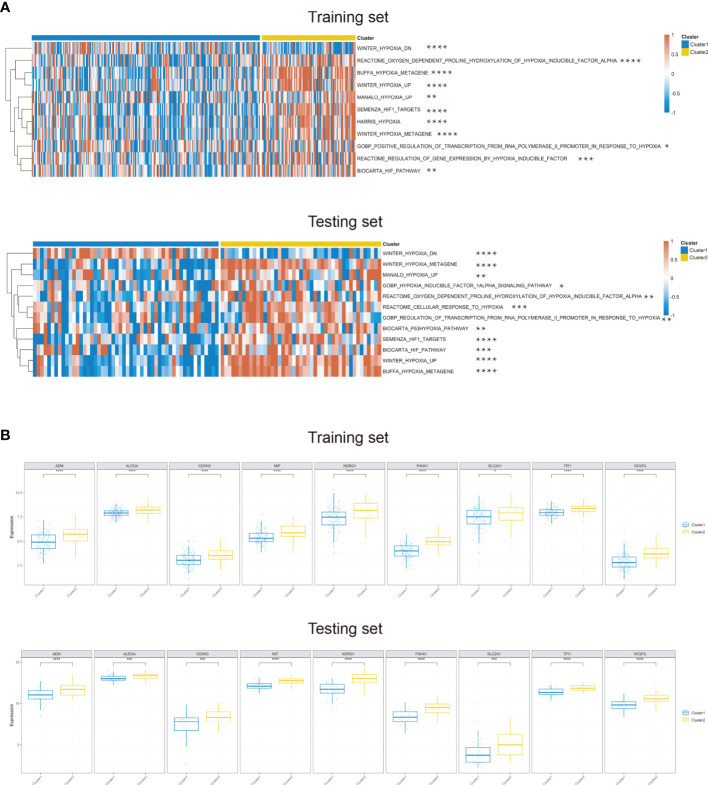
Differential hypoxia conditions across two identified clusters. **(A)** Heatmap of the significant differential hypoxia pathways of two OSCC clusters based on ssGSEA in the training set and testing set. **(B)** The expression of nine hypoxia key genes upregulated in cluster 2 in the training and testing sets (*P < 0.05, **P < 0.01, ***P < 0.001, ****P < 0.0001).

To better understand the hypoxia status of the two clusters, we conducted the ssGSEA algorithm to calculate the scores of some hypoxia-associated processes. As expected, patients in cluster 2 were enriched in higher hypoxia condition in the training and testing cohorts (**Figure 2A**). What is more, a total of nine hypoxia-associated key genes were also verified to be highly expressed in cluster 2, which was consistent with the aforesaid ssGSEA result (**Figure 2B**). Hence, we could define cluster 2 as a “high hypoxia subclass” compared with cluster 1.

### Mutation Alterations in the Two Subclasses

**Figure 3:**
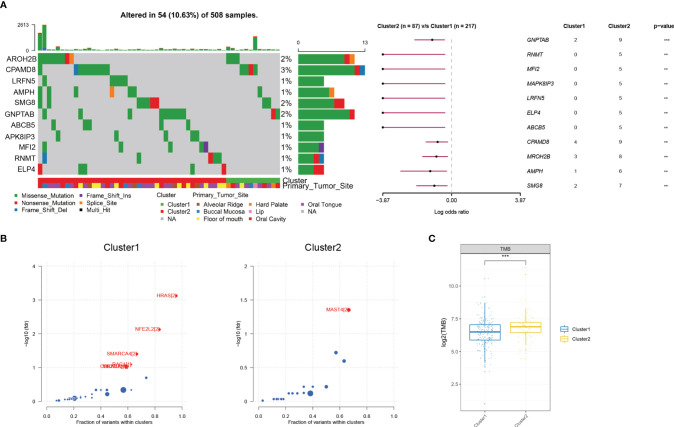
Mutational alterations between two hypoxia clusters in the TCGA cohort. **(A)** Top 11 most differently mutated genes depicted in the two clusters. **(B)** *HRAS* or *MAST4* respectively identified as the driver gene candidate for cluster 1 or cluster 2. **(C)** Tumor mutational burden significantly increased in cluster 2 (**P < 0.01, ***P < 0.001).

Recent studies have reported the hypoxia phenotype associated with gene mutations (30). We further investigated the difference of gene mutations among these two clusters. As illustrated in the waterfall plot, differently mutated genes were detected between the two clusters and *GNPTAB* was finally identified as the most differentially highly mutated gene in cluster 2 (**Figure 3A**) (*P* < 0.01). Furthermore, based on the oncodriveCLUST algorithm, we predicted *HRAS* as the driver gene candidate in cluster 1 and *MAST4* in cluster 2 (**Figure 3B**). What is more, tumor mutational burden (TMB) was significantly increased in cluster 2 (**Figure 3C**).

### High Correlation Between Hypoxia-Related Gene-Based Clusters With Immune Infiltration

To obtain deeper insights into the molecular characteristics of the two OSCC clusters, we conducted the differentially expressed genes (DEGs) analysis and their GO analysis in the training dataset. With a threshold of |log2 FC| >1.2 and adjusted *P*-value <0.05, a sum of 55 DEGs were identified for the two clusters. The expressions of DEG between these two clusters were demonstrated by a heatmap (**Figure S2A**). GO analysis based on Cytoscape showed that the cluster-specific genes were significantly enriched in immune cell infiltration, suggesting a distinct immune difference between these two clusters (**Figure S2B**).

### Immune Microenvironment Features Between the Two Clusters

**Figure 4:**
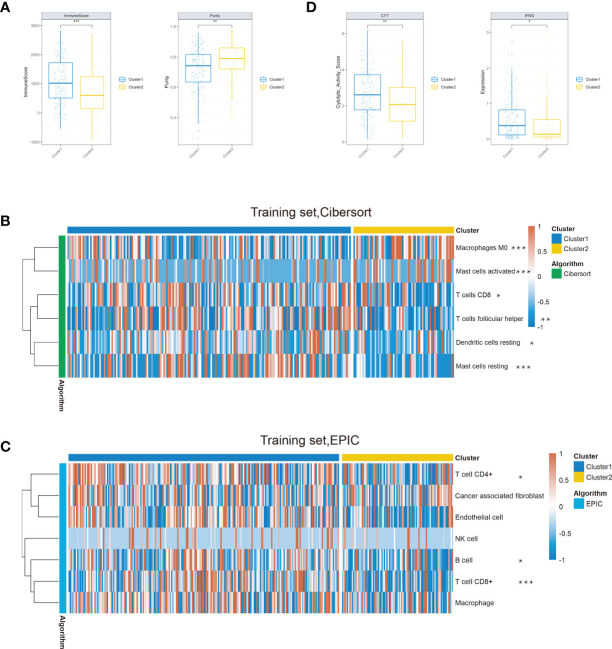
Comparison of the immune conditions and TME between the two clusters in the training set. **(A)** Cluster 2 occupied a lower immune score and a higher purity than cluster 1. **(B)** Composition of the six significantly differential immune cells between the two clusters based on the CIBERSORT algorithm. **(C)** The Epic algorithm illustrated the immune cell difference between the two clusters. **(D)** The CYT score and IFNG expression significantly decreased in cluster 2 (*P < 0.05, **P < 0.01, ***P < 0.001).

To reveal the difference of these two clusters on the tumor microenvironment, we first calculated the immune score and tumor purity both in the training and testing sets based on the ESTIMATE algorithm. We found that the immune score was decreased and purity score was elevated in cluster 2 compared with cluster 1 (**Figures 4A** and **S3A**). With the significant difference in immune score and purity score identified between clusters, we further compared the relative ratio of 22 kinds of immune cells by the CIBERSORT algorithm. There existed six immune cell populations significantly differently enriched between the two clusters in the training set and nine immune cells in the testing set (**Figures 4B** and **S3B**). Combined, macrophages M0, activated mast cells, were enriched in cluster 2, while CD8 T cells, resting mast cells, were deficient in both two sets. We further conducted the Epic algorithm to validate our results and found that only CD8 T cells were consistently lacking in cluster 2 in the two cohorts (**Figures 4C** and **S3C**). CD8 T cell, also known as cytotoxic T cell (CTL), exerted a critical role in antitumor immunity. We further examined two indicators of T-cell killing ability between the two clusters. Similarly, cluster 2 also exhibited lower CYT score and IFNG expression than cluster 1 in the training set and testing set, which was consistent with previous studies that showed an association between high CYT levels and higher patient OS (**Figures 4D** and **S3D**). Taken together, it was the lower composition of CD8 T cells and their disability of killing tumor cells that led a worse prognosis in cluster 2.

### Identification of the Potential Treatment Strategy of the Two Clusters

**Figure 5:**
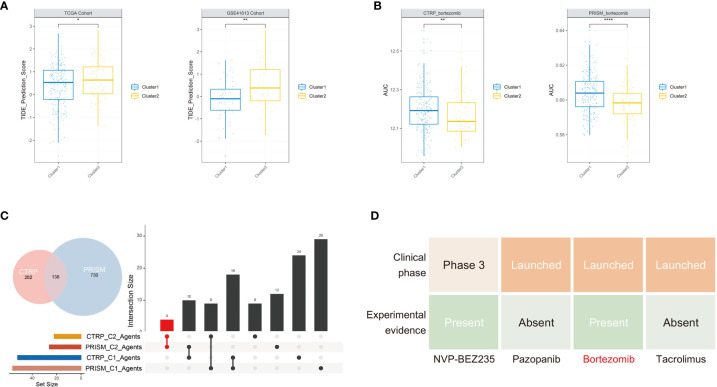
Potential treatment strategy of the two clusters. **(A)** Cluster 2 group occupied a significantly higher TIDE score in the two cohorts. **(B)** The predicted AUC values of bortezomib from the CTRP and PRISM cohorts were decreased in cluster 2 patients. **(C)** The upsetR plot revealed only the AUC of four agents simultaneously decreased in cluster 2 patients estimated by the CTRP and PRISM cohorts. **(D)** Identification of the most promising cluster 2-specific agents according to evidence from multiple sources (*P < 0.05, **P < 0.01, ****P < 0.0001).

After investigating the distinct molecular and biological characters between these two clusters, we sought to explore specific treatment options for each cluster. Considering the vital role of CD8 T cells in immunotherapy and their significant differences between the two clusters, we further assessed their immunotherapy response based on the TIDE method. In both training set and testing set, the TIDE score was significantly lower in cluster 1 compared with cluster 2, indicating patients in cluster 1 might be more sensitive to ICB therapy (**Figure 5A**). For cluster 2 patients, we hoped to seek for traditional chemotherapeutics to achieve targeted therapy. After the filtering procedure described in the *Material and Methods*, we finally obtained 16 OSCC cells with 913 drugs in the PRISM and 22 OSCC cells with 465 drugs in the CTRP dataset. The pRRophetic package with a built-in ridge regression model was then applied to predict the drug response of clinical samples in the training set based on their expression profiles, and the estimated AUC value of each compound in each sample was thus obtained. We finally identified four agents simultaneously with lower AUC values in cluster 2 both in the PRISM- and CTRP-predicted datasets (**Figures 5B, C** and **S4**). To further filter a more therapeutically significant drug in OSCC, we took their clinical phase and experimental evidence from the literature into account. Finally, we identified only bortezomib as the optimal drug that has the potential for cluster 2 treatment (**Figure 5D**).

### Development and Validation of Hypoxia-Associated Prognostic Signature

**Figure 6:**
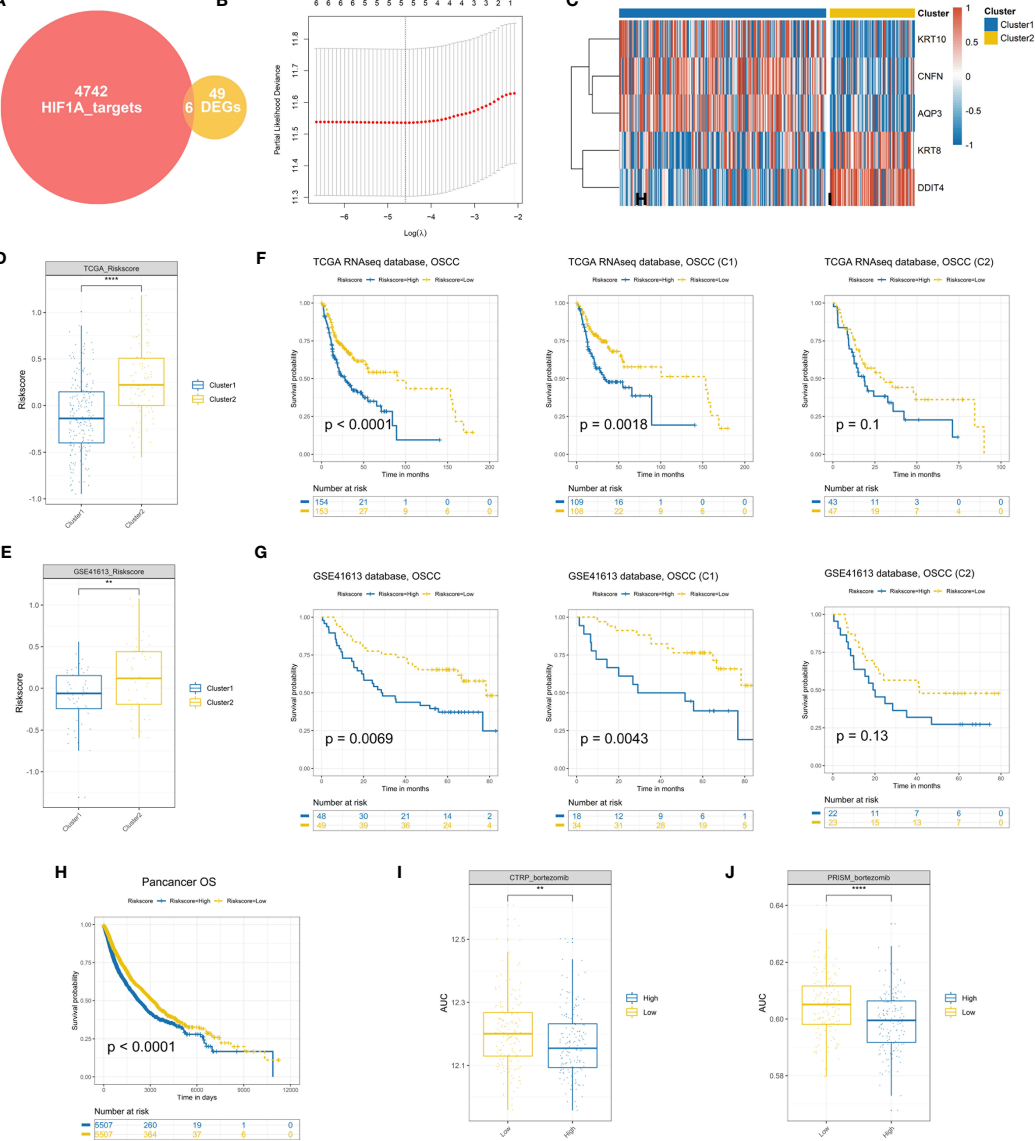
Establishment and validation of a hypoxia prognostic signature **(A)**. A total of six candidate genes were identified in the intersection of “HIF1A targets” and “DEGs.” **(B)** Cross-validation for tuning parameter selection in the proportional hazards model. **(C)** Differential expression of genes in hypoxia signature. **(D, E)** Cluster 2 patients conferred a significantly higher hypoxia risk scores in the TCGA and GSE41613 cohorts. **(F, G)** Survival analysis of the hypoxia-associated signature in OSCC or OSCC subtypes. **(H)** The prognostic significance of the established signature across 33 cancer types. **(I, J)** The predicted AUC values of bortezomib from the CTRP and PRISM datasets were decreased in the high-risk score OSCC patients (**P < 0.01, ****P < 0.0001).

To establish a signature for clinical implications, it is of great significance to filter the most representative genes of each cluster. Considering HIF1A serving as the key transcription factor in hypoxia, we intersected the DEGs between the two clusters with 4,748 potential targets of HIF1A in OSCC and found a sum of 6 candidate genes in the intersection (**Figure 6A**), identified as “Clustering-specific hypoxia-related genes.” To obtain the most powerful prognostic markers, the LASSO Cox regression analysis was conducted (**Figure 6B**). A total of five gene signatures were generated and the coefficients were estimated by multivariate Cox regression *via* LASSO (**Table S2**). There existed a transcriptional difference between the two clusters (**Figure 6C**). After calculating the risk scores of the signature based on the regression coefficients, we intriguingly found that cluster 2 possessed a higher score in the two cohorts (**Figures 6D, E**). Further survival analysis revealed that patients in the high-score group exhibited significantly worse prognosis than OSCC patients or cluster 1 patients with low-score (**Figures 6F, G**). Although there was no significant survival difference between high and low scores in cluster 2 in the training set (*P* = 0.1) and testing set (*P* = 0.13), it was still obvious that a high hypoxia score was associated with the tendency toward worse prognosis (**Figures 6F, G**). The results were consistent with the above data that cluster 2 conferred the poorer prognosis. In order to determine the prognostic significance of the signature in other organ sites, we conducted the survival analysis of our hypoxia score across 33 TCGA cancer types. Similarly, the hypoxia risk score also served as an unfavorable prognostic biomarker for pancancer (**Figure 6H**). What is more, the predicted AUC values of bortezomib from CTRP and PRISM were also decreased in the high hypoxia score group, validating its promising clinical value for high-risk OSCC patients (**Figures 6I, J**).

### Construction of a Nomogram for Predicting OSCC Survival

**Figure 7:**
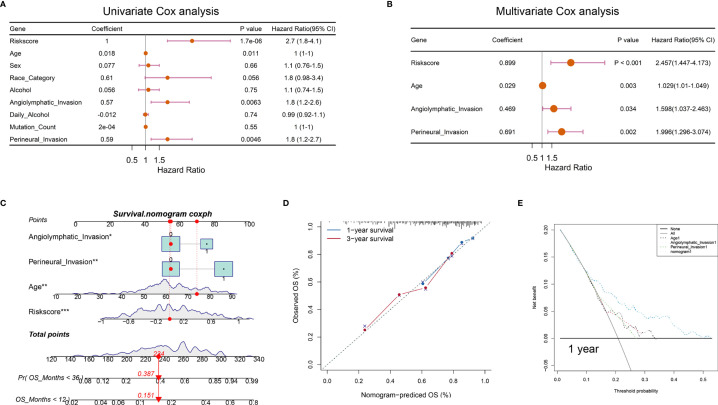
Nomograms according to the OS-associated hypoxia scores for OSCC patients in the TCGA cohort. **(A, B)** The univariate and multivariate Cox regression analyses of OS-associated variables. **(C)** Establishment of a nomogram to predict the OS of OSCC patients. **(D)** The calibration curve revealed the high consistency between the nomogram-predicted OS with actual OS. **(E)** Decision curve analysis for the nomogram and other clinical features in the prediction of prognosis of OSCC patients at 1-year point (*P < 0.05, **P < 0.01, ***P < 0.001).

To verify whether the hypoxia-related signature was an independent prognostic factor, univariate and multivariate Cox regression analyses were conducted (**Figures 7A, B**). The results in univariate Cox regression revealed that risk score, age, and angiolymphatic and perineural invasion had a significant association with the OS of OSCC patients. In multivariate Cox regression, risk score, age, and angiolymphatic and perineural invasion were identified as independent prognostic factors of OSCC. Then, we applied these four independent factors to establish a nomogram for predicting OSCC 1- and 3-year OS (**Figure 7C**). With the score increasing, the OS of patients decreased. Moreover, the calibration plots at 1 and 3 years approached 45 degrees, indicating a great performance of the established nomogram (**Figure 7D**). Meanwhile, DCA was performed to compare the clinical usability and benefits of the nomogram with that of the age and angiolymphatic and perineural invasion. As shown in **Figure 7E**, compared with age and angiolymphatic and perineural invasion, the 1-year DCA curves of the new nomogram showed larger net benefits across a range of death risk.

### Predictive Value of Hypoxia-Related Risk Score in Immunotherapy

**Figure 8:**
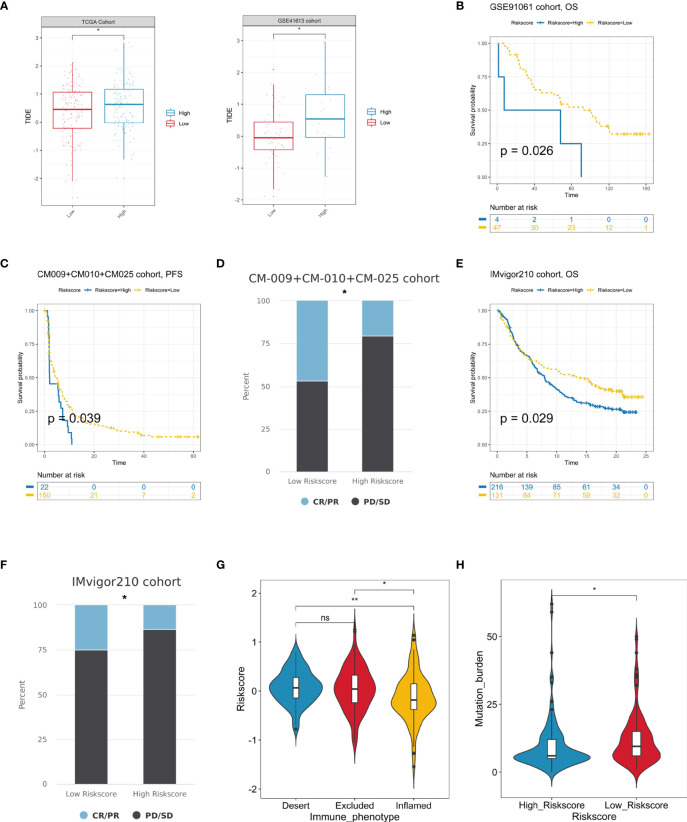
Prediction of immunotherapeutic benefits by hypoxia score. **(A)** TIDE scores were increased in the high hypoxia risk score group in the TCGA and GSE41613 cohorts. **(B, C)** The survival analysis of the high and low hypoxia risk score groups in the GSE91061 and CM-009+CM-010+CM025 immunotherapy cohorts. **(D)** The immunotherapy response patients (CR/PR) more distributed in lower risk score patients, while non-response ones (PD/SD) enriched in higher risk score patients in the CM-009+CM-010+CM025 cohorts. **(E)** High-risk score patients occupied a significantly reduced overall survival in the Imvigor210 cohort. **(F)** Various fractions of clinical outcome patients in the high and low hypoxia risk score groups in the IMvigor210 cohort. **(G)** The difference of hypoxia risk scores in the three immune subtype groups in the IMvigor210 cohort. **(H)** Differences in TMB between high- and low-risk score groups in the IMvigor210 cohort (ns, no significance, *P < 0.05, **P < 0.01).

Immunotherapy has been proven relevant to improve survival in the treatment of multiple tumor types. Thus, identification of patients who will benefit most from ICB treatment is of great necessity. Our analysis revealed that the TIDE was significantly increased in the high hypoxia score group, indicating its crucial role in regulating immune response (**Figure 8A**). Based on three immunotherapy cohorts, we identified that patients with a high hypoxia score group always exhibited clinical disadvantages and markedly shortened survival (*P* = 0.026 in GSE91061, *P* = 0.039 in CM009+010+025 cohorts, and *P* = 0.029 in IMvigor210) (**Figures 8B, C, E**). In CM009+010+025 cohorts, the chi-squared test conducted between low and high hypoxia score groups demonstrated significantly better therapeutic outcomes in low score patients (**Figure 8D**). Similarly, patients with high hypoxia scores exhibited less treatment effectiveness in the IMvigor210 cohort (**Figure 8F**). We also compared the hypoxia score levels in the three immune subtypes of IMvigor210. The immune-inflamed subtype showed significantly the lowest risk score, which further confirmed our analysis above (**Figure 8G**). In addition, TMB was significantly decreased in the high-score group (**Figure 8H**). In all, our results strongly suggested that hypoxia score was associated with the response to immunotherapy and could further effectively predict the prognosis of patients.

## Discussion

The tumor microenvironment is composed of not only the solid tumor tissue but also the surrounding vessels, fibroblasts, distinct immune cells, and extracellular matrix (31, 32). The imbalance between excessive oxygen demand and insufficient oxygen supply shaped a hypoxic microenvironment, leading to a malignant progression of tumor (33). As a hallmark of tumor, hypoxia exerts a crucial significance in different biological processes, including multiple metabolic forms, immune escape, angiogenesis, and metastasis (34). What is more, the crosstalk between tumor cells and other non-tumor cells under a hypoxic microenvironment could also induce therapeutic resistance, resulting in failure of treatment and poor clinical outcome. Considering hypoxia as an emerging biomarker and target in cancer therapy, exploring the effect of hypoxia in the tumor microenvironment is of great necessity.

Up till now, more and more studies emphasize the importance of molecular subtyping, which could direct individualized treatment (35, 36). The classification based on hypoxia genes and the generation of related signatures have been conducted in many cancer types including breast cancer, lung adenocarcinoma, and glioma to discriminate high-risk subclass and to predict survival (21, 37, 38). However, the relationships between hypoxia with clinical outcomes, genomic alterations, and therapeutic responses remain obscure in OSCC. Identifying different hypoxia patterns and generating a related signature in OSCC are beneficial to deepen our understanding of hypoxic microenvironment in OSCC progression and improve the outcome of cancer treatment.

In our study, we recognized two hypoxia-associated patterns that have different characteristics by unsupervised clustering of the gene expression of hypoxia genes. Cluster 2 patients were characterized by higher hypoxia degree, leading to a survival disadvantage over cluster 1. We also explored different mutated patterns between the two clusters. Moreover, we identified hypoxia signature genes by conducting differentially expressed analysis between the two subtypes. In agreement with the association of hypoxia status with abnormal immune response, we found that the signature genes were correlated with distinct immune cell infiltration. In the tumor microenvironment (TME), CD8 + CTLs are the immune cells of first choice for targeting cancer. During cancer progression, CTL encounters dysfunction and exhaustion due to immune-related tolerance and immunosuppression in TME, all of which contribute to adaptive immune resistance. Through multiple algorithms in the two databases, we identified CD8 T cells consistently deficient in cluster 2, which might be a major cause of its poorer prognosis and its worse immunotherapy response.

Thinking of the heterogeneity of hypoxia conditions, it was essential to quantify the hypoxia-associated character in OSCC. Hence, we further established a hypoxia-related scoring system and validated it in two cohorts. The estimated risk score was elevated in cluster 2, which was consistent with its worse prognostic significance. Multivariate Cox analysis also revealed the score as an independent prognostic factor in OSCC. Furthermore, the predictive potential of this prognostic risk score model was generated by combining it with several clinical features in a risk assessment nomogram.

In view of the clinical significance of our study, we respectively investigated different treatment strategies for distinct subclasses in line with the concept of precision treatment. For cluster 1 with a better prognosis, we recommended the recently widely used ICB treatment, while for cluster 2 patients, we screened bortezomib as the promising agent to improve the outcome of this subtype. What is more, the ideal drug was also applied to OSCC patients with high hypoxia-related risk score, indicating its clinical transforming value. In addition, the risk score we established could also predict the efficacy of immune checkpoint therapy and might promote personalized OSCC immunotherapy in future ICB treatment.

In summary, we recognized two different subclasses with a distinct immune microenvironment in OSCC based on hypoxia condition and explored the treatment of each subtype. We also established an individual hypoxia-associated score system which could predict the survival and the efficacy of immunotherapy. These findings provide a novel, efficient, and accurate predictive model in the prognosis and response to immunotherapy, thus promoting personalized cancer chemotherapy and immunotherapy in the future.

## Data Availability Statement

The original contributions presented in the study are included in the article/**Supplementary Material**. Further inquiries can be directed to the corresponding author.

## Author Contributions

Concept and design: CL and ZZ. Data download and curation: XC, XR, JC, HC, JY, XMC, and SK. Data analyses: CL, XC, XR, and QR. Manuscript writing and revision: CL, XC, XR, and ZZ. All authors contributed to the article and approved the submitted version.

## Conflict of Interest

The authors declare that the research was conducted in the absence of any commercial or financial relationships that could be construed as a potential conflict of interest.

## Publisher’s Note

All claims expressed in this article are solely those of the authors and do not necessarily represent those of their affiliated organizations, or those of the publisher, the editors and the reviewers. Any product that may be evaluated in this article, or claim that may be made by its manufacturer, is not guaranteed or endorsed by the publisher.

## Supplementary Material

Supplementary Figure 1 Screening of the significantly prognostic hypoxia genes in TCGA cohort. **(A)** Univariate Cox regression analysis of 200 classical hypoxia stimulated genes in TCGA cohort (The forest plot only illustrated the significant genes).

Supplementary Figure 2 Identification of the DEGs and related functions between two clusters in TCGA cohorts. **(A)** The DEGs heatmap between two clusters in OSCC. **(B)** Cluego illustrated a high correlation with immune cells infiltration.

Supplementary Figure 3 The TME characteristics between two clusters in GSE41613 cohort. **(A)** Cluster2 patients conferred lower immune scores and higher tumor purity than Cluster1. **(B)** The significant immune cells difference between two clusters estimated by Cibersort algorithm. **(C)** EPIC algorithm revealed 7 immune cells difference in two clusters. **(D)** The CYT score but not the IFNG expression significantly decreased in Cluster2 patients. (*P < 0.05, **P < 0.01, ***P < 0.001, ****P < 0.0001, NS, not significant)

Supplementary Figure 4 Compare of two Clusters’ predicted AUC of another three drugs in TCGA cohorts. **(A)** The AUC values of another three agents predicted from CTRP and PRISM **(B)** were significantly decreased in Cluster2 patients. **P < 0.01, ***P < 0.001, ****P < 0.0001)
