# Supplementary figures and images for: Identification of Hypoxia-Related Molecular Classification and Associated Gene Signature in Oral Squamous Cell Carcinoma
Source: Front Oncol. 2021 Nov 23;11:709865. doi: 10.3389/fonc.2021.709865 (PMC8649955; doi:10.3389/fonc.2021.709865)

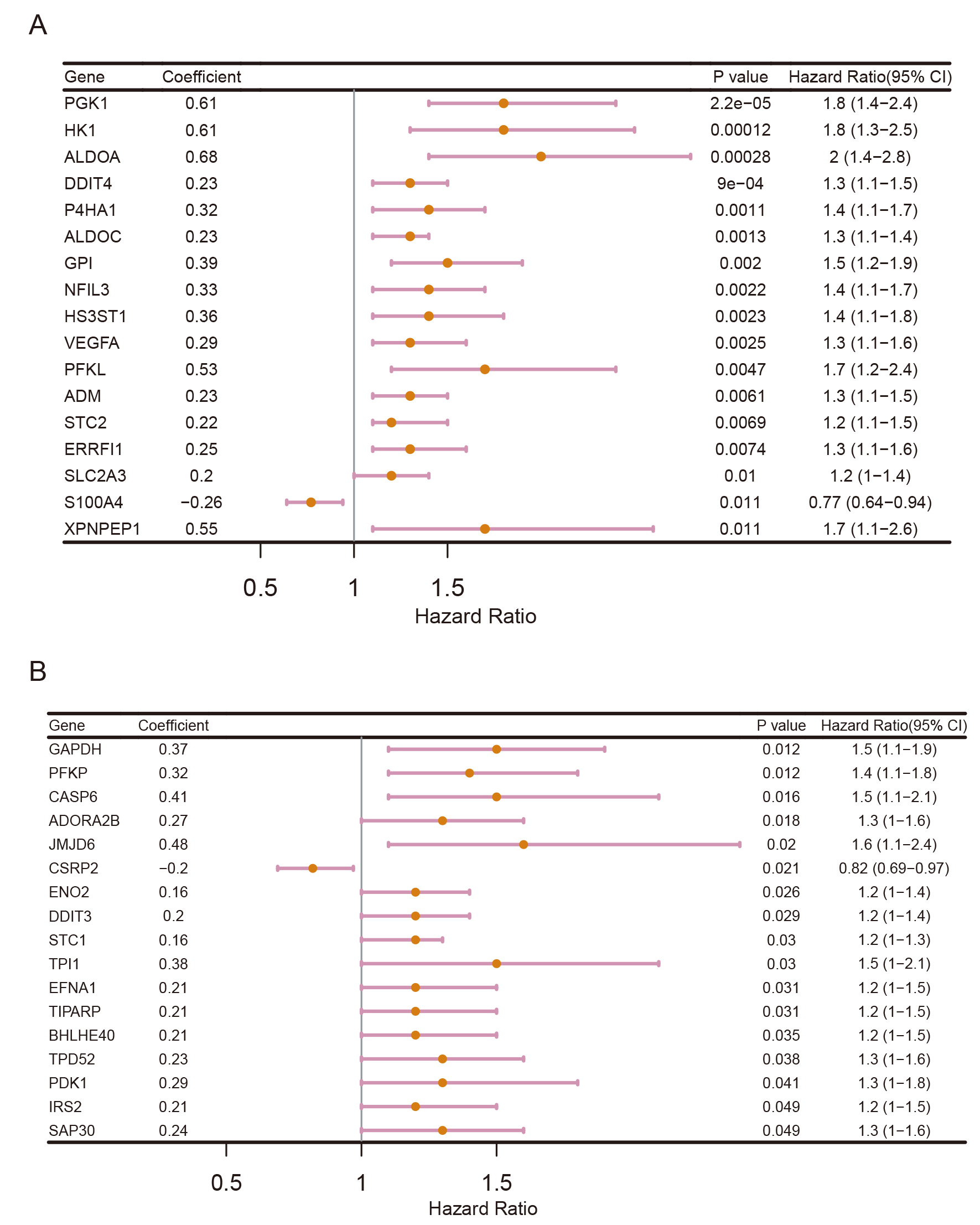

Supplement: Supplementary Figure 1 — Screening of the significantly prognostic hypoxia genes in TCGA cohort. (A) Univariate Cox regression analysis of 200 classical hypoxia stimulated genes in TCGA cohort (The forest plot only illustrated the significant genes). [file Image_1.tif]

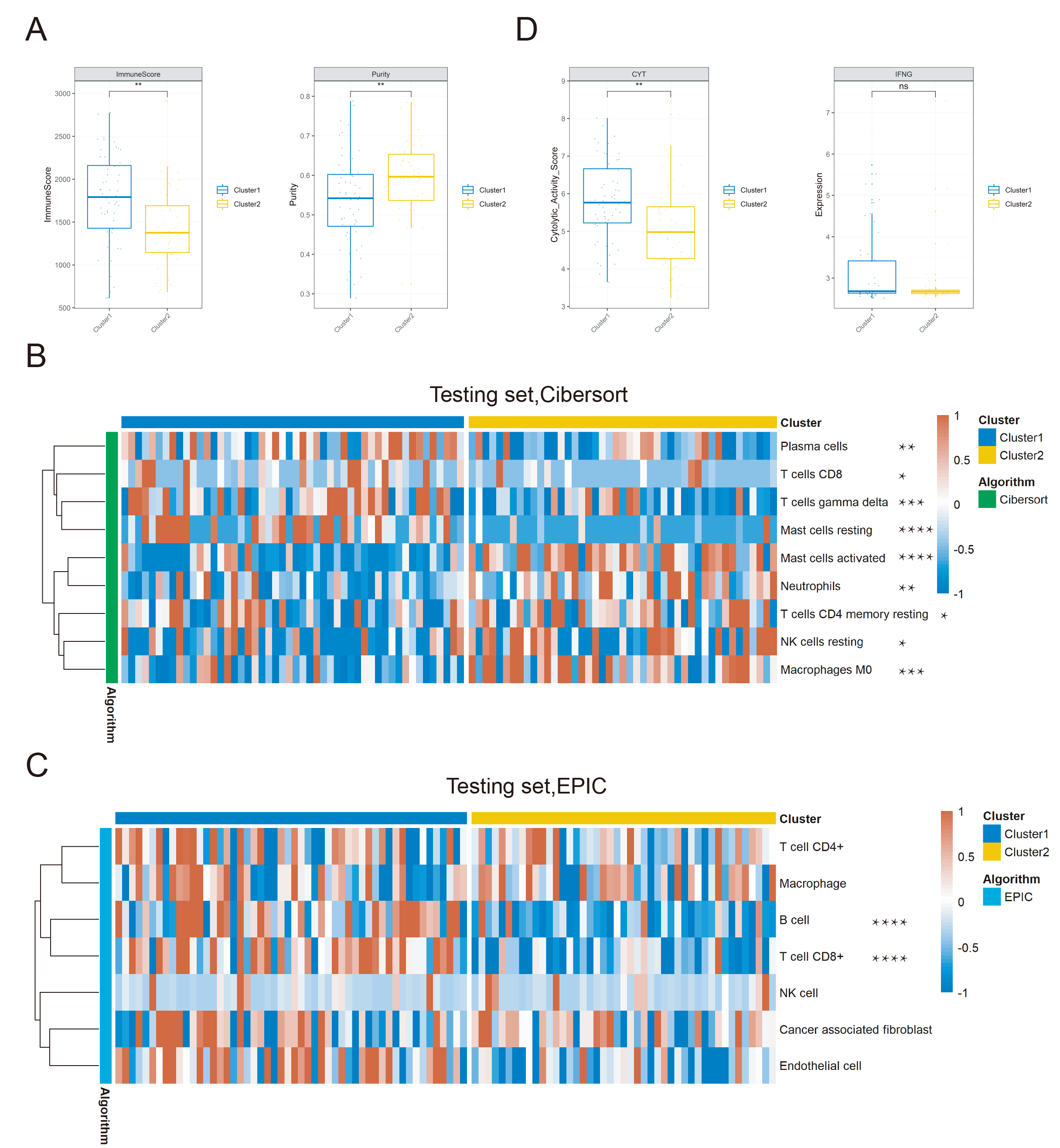

Supplement: Supplementary Figure 3 — The TME characteristics between two clusters in GSE41613 cohort. (A) Cluster2 patients conferred lower immune scores and higher tumor purity than Cluster1. (B) The significant immune cells difference between two clusters estimated by Cibersort algorithm. (C) EPIC algorithm revealed 7 immune cells difference in two clusters. (D) The CYT score but not the IFNG expression significantly decreased in Cluster2 patients. (*P < 0.05, **P < 0.01, ***P < 0.001, ****P < 0.0001, NS, not significant) [file Image_3.tif]

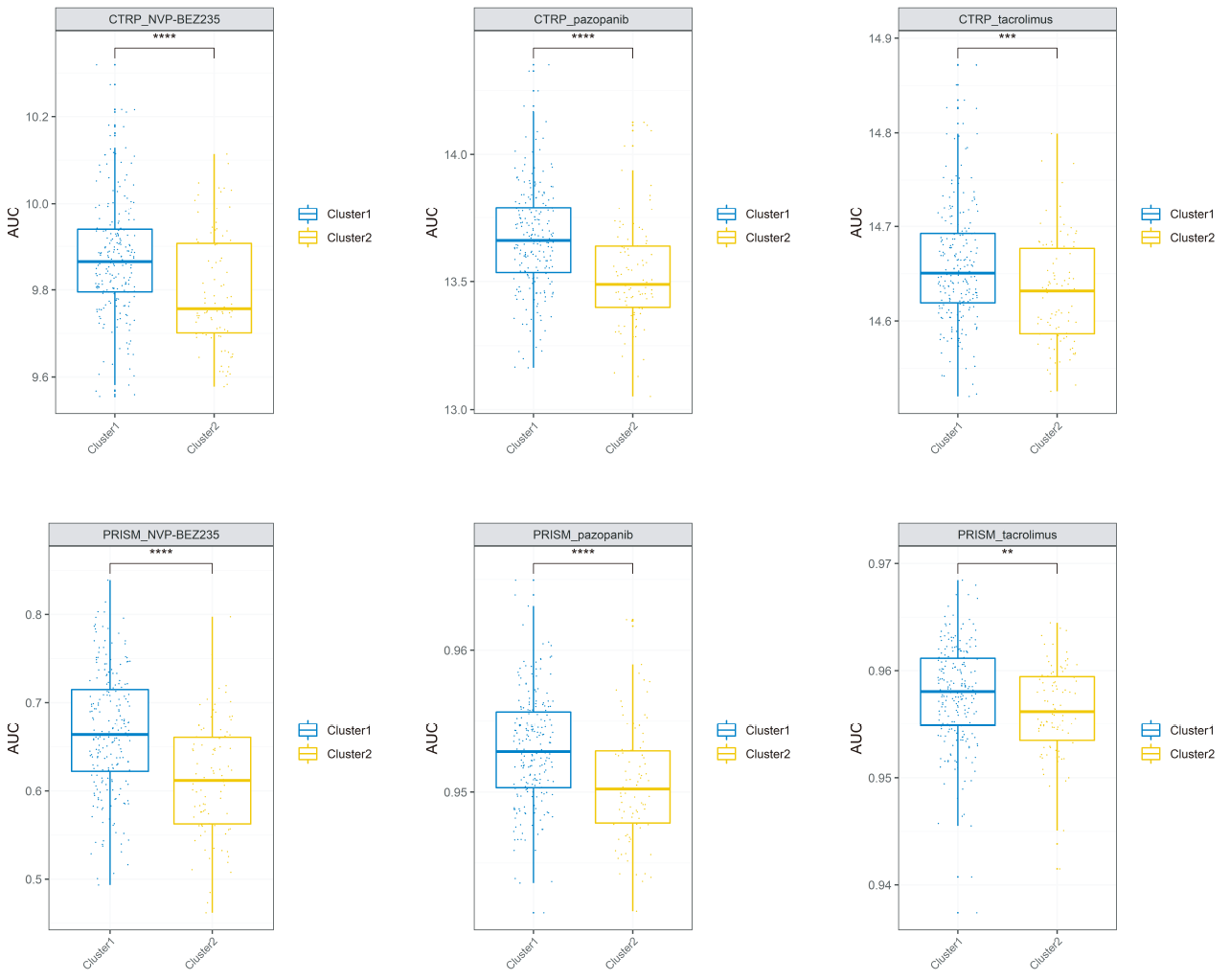

Supplement: Supplementary Figure 4 — Compare of two Clusters’ predicted AUC of another three drugs in TCGA cohorts. (A) The AUC values of another three agents predicted from CTRP and PRISM (B) were significantly decreased in Cluster2 patients. **P < 0.01, ***P < 0.001, ****P < 0.0001) [file Image_4.tif]
