# Supplementary material for: Identification of Hypoxia-Related Molecular Classification and Associated Gene Signature in Oral Squamous Cell Carcinoma
Source: Front Oncol. 2021 Nov 23;11:709865. doi: 10.3389/fonc.2021.709865 (PMC8649955; doi:10.3389/fonc.2021.709865)
Supplement: Supplementary file 5 [file Table_1.docx]

Table S1: Univariate cox analysis of classical hypoxia stimulated genes.

| [HALLMARK_](http://www.gsea-msigdb.org/gsea/msigdb/cards/HALLMARK_APOPTOSIS.html)  [HYHPOXIA](http://www.gsea-msigdb.org/gsea/msigdb/cards/HALLMARK_APOPTOSIS.html) | beta | HR (95% CI for HR) | wald.test | p.value |
| --- | --- | --- | --- | --- |
| PGK1 | 0.61 | 1.8 (1.4-2.4) | 18 | 2.20E-05 |
| HK1 | 0.61 | 1.8 (1.3-2.5) | 15 | 0.00012 |
| ALDOA | 0.68 | 2 (1.4-2.8) | 13 | 0.00028 |
| DDIT4 | 0.23 | 1.3 (1.1-1.5) | 11 | 9.00E-04 |
| P4HA1 | 0.32 | 1.4 (1.1-1.7) | 11 | 0.0011 |
| ALDOC | 0.23 | 1.3 (1.1-1.4) | 10 | 0.0013 |
| GPI | 0.39 | 1.5 (1.2-1.9) | 9.5 | 0.002 |
| NFIL3 | 0.33 | 1.4 (1.1-1.7) | 9.4 | 0.0022 |
| HS3ST1 | 0.36 | 1.4 (1.1-1.8) | 9.3 | 0.0023 |
| VEGFA | 0.29 | 1.3 (1.1-1.6) | 9.2 | 0.0025 |
| PFKL | 0.53 | 1.7 (1.2-2.4) | 8 | 0.0047 |
| ADM | 0.23 | 1.3 (1.1-1.5) | 7.5 | 0.0061 |
| STC2 | 0.22 | 1.2 (1.1-1.5) | 7.3 | 0.0069 |
| ERRFI1 | 0.25 | 1.3 (1.1-1.6) | 7.2 | 0.0074 |
| SLC2A3 | 0.2 | 1.2 (1-1.4) | 6.6 | 0.01 |
| S100A4 | -0.26 | 0.77 (0.64-0.94) | 6.4 | 0.011 |
| XPNPEP1 | 0.55 | 1.7 (1.1-2.6) | 6.4 | 0.011 |
| GAPDH | 0.37 | 1.5 (1.1-1.9) | 6.3 | 0.012 |
| PFKP | 0.32 | 1.4 (1.1-1.8) | 6.3 | 0.012 |
| CASP6 | 0.41 | 1.5 (1.1-2.1) | 5.8 | 0.016 |
| ADORA2B | 0.27 | 1.3 (1-1.6) | 5.6 | 0.018 |
| JMJD6 | 0.48 | 1.6 (1.1-2.4) | 5.4 | 0.02 |
| CSRP2 | -0.2 | 0.82 (0.69-0.97) | 5.3 | 0.021 |
| ENO2 | 0.16 | 1.2 (1-1.4) | 5 | 0.026 |
| DDIT3 | 0.2 | 1.2 (1-1.4) | 4.8 | 0.029 |
| STC1 | 0.16 | 1.2 (1-1.3) | 4.7 | 0.03 |
| TPI1 | 0.38 | 1.5 (1-2.1) | 4.7 | 0.03 |
| EFNA1 | 0.21 | 1.2 (1-1.5) | 4.6 | 0.031 |
| TIPARP | 0.21 | 1.2 (1-1.5) | 4.6 | 0.031 |
| BHLHE40 | 0.21 | 1.2 (1-1.5) | 4.4 | 0.035 |
| TPD52 | 0.23 | 1.3 (1-1.6) | 4.3 | 0.038 |
| PDK1 | 0.29 | 1.3 (1-1.8) | 4.2 | 0.041 |
| IRS2 | 0.21 | 1.2 (1-1.5) | 3.9 | 0.049 |
| SAP30 | 0.24 | 1.3 (1-1.6) | 3.9 | 0.049 |
| PKLR | -1.2 | 0.31 (0.097-1) | 3.8 | 0.052 |
| ENO1 | 0.33 | 1.4 (0.99-2) | 3.6 | 0.058 |
| NAGK | -0.32 | 0.73 (0.53-1) | 3.6 | 0.058 |
| HSPA5 | 0.3 | 1.4 (0.98-1.9) | 3.4 | 0.063 |
| MIF | 0.2 | 1.2 (0.99-1.5) | 3.4 | 0.067 |
| AMPD3 | -0.25 | 0.78 (0.59-1) | 3.3 | 0.068 |
| CA12 | 0.16 | 1.2 (0.99-1.4) | 3.3 | 0.068 |
| ISG20 | -0.17 | 0.85 (0.71-1) | 3.3 | 0.068 |
| HEXA | 0.31 | 1.4 (0.97-1.9) | 3.2 | 0.073 |
| NCAN | 1.1 | 3.1 (0.88-11) | 3.1 | 0.078 |
| ATP7A | 0.3 | 1.3 (0.96-1.9) | 3 | 0.081 |
| EGFR | 0.12 | 1.1 (0.99-1.3) | 3 | 0.082 |
| HOXB9 | 0.17 | 1.2 (0.98-1.4) | 3 | 0.083 |
| GCNT2 | 0.23 | 1.3 (0.97-1.6) | 2.9 | 0.09 |
| TNFAIP3 | 0.14 | 1.1 (0.98-1.3) | 2.9 | 0.091 |
| SIAH2 | 0.23 | 1.3 (0.96-1.6) | 2.8 | 0.094 |
| CHST2 | -0.11 | 0.89 (0.78-1) | 2.7 | 0.098 |
| MXI1 | 0.23 | 1.3 (0.95-1.7) | 2.7 | 0.1 |
| ANXA2 | 0.27 | 1.3 (0.94-1.8) | 2.6 | 0.11 |
| IER3 | 0.13 | 1.1 (0.97-1.3) | 2.6 | 0.11 |
| MAFF | 0.2 | 1.2 (0.95-1.6) | 2.5 | 0.11 |
| NDST1 | -0.19 | 0.83 (0.65-1) | 2.5 | 0.11 |
| NEDD4L | 0.21 | 1.2 (0.96-1.6) | 2.6 | 0.11 |
| DTNA | 0.25 | 1.3 (0.93-1.7) | 2.4 | 0.12 |
| GPC1 | 0.17 | 1.2 (0.96-1.5) | 2.4 | 0.12 |
| SLC25A1 | 0.21 | 1.2 (0.95-1.6) | 2.4 | 0.12 |
| TGFB3 | -0.13 | 0.88 (0.75-1) | 2.4 | 0.12 |
| SDC4 | 0.16 | 1.2 (0.96-1.4) | 2.3 | 0.13 |
| LDHA | 0.18 | 1.2 (0.94-1.5) | 2.2 | 0.14 |
| IDS | 0.2 | 1.2 (0.93-1.6) | 2.1 | 0.15 |
| KDM3A | 0.21 | 1.2 (0.93-1.6) | 2.1 | 0.15 |
| PYGM | 0.084 | 1.1 (0.97-1.2) | 2.1 | 0.15 |
| EDN2 | 0.077 | 1.1 (0.97-1.2) | 1.9 | 0.16 |
| GYS1 | 0.26 | 1.3 (0.9-1.9) | 2 | 0.16 |
| TPBG | 0.2 | 1.2 (0.93-1.6) | 2 | 0.16 |
| SCARB1 | 0.16 | 1.2 (0.93-1.5) | 1.9 | 0.17 |
| ATF3 | 0.082 | 1.1 (0.96-1.2) | 1.8 | 0.18 |
| GPC3 | 0.069 | 1.1 (0.97-1.2) | 1.8 | 0.18 |
| PRKCA | 0.2 | 1.2 (0.91-1.6) | 1.8 | 0.18 |
| WSB1 | 0.19 | 1.2 (0.92-1.6) | 1.8 | 0.18 |
| CDKN1B | 0.2 | 1.2 (0.91-1.6) | 1.7 | 0.19 |
| PPP1R15A | 0.15 | 1.2 (0.93-1.5) | 1.8 | 0.19 |
| GRHPR | 0.2 | 1.2 (0.9-1.6) | 1.7 | 0.2 |
| SLC2A1 | 0.097 | 1.1 (0.95-1.3) | 1.6 | 0.2 |
| TES | 0.18 | 1.2 (0.91-1.6) | 1.6 | 0.2 |
| VHL | 0.2 | 1.2 (0.9-1.6) | 1.6 | 0.2 |
| NDRG1 | 0.099 | 1.1 (0.95-1.3) | 1.6 | 0.21 |
| ACKR3 | 0.077 | 1.1 (0.96-1.2) | 1.5 | 0.22 |
| FAM162A | 0.14 | 1.1 (0.92-1.4) | 1.5 | 0.22 |
| TGM2 | 0.076 | 1.1 (0.95-1.2) | 1.5 | 0.22 |
| B4GALNT2 | -0.22 | 0.81 (0.56-1.2) | 1.4 | 0.25 |
| ENO3 | 0.063 | 1.1 (0.96-1.2) | 1.3 | 0.25 |
| EXT1 | 0.14 | 1.1 (0.9-1.5) | 1.2 | 0.27 |
| KLHL24 | 0.11 | 1.1 (0.92-1.3) | 1.2 | 0.27 |
| SRPX | 0.077 | 1.1 (0.94-1.2) | 1.2 | 0.27 |
| F3 | 0.059 | 1.1 (0.95-1.2) | 1.2 | 0.28 |
| GBE1 | 0.16 | 1.2 (0.88-1.6) | 1.2 | 0.28 |
| AKAP12 | 0.1 | 1.1 (0.91-1.3) | 1.1 | 0.29 |
| FOS | -0.062 | 0.94 (0.84-1.1) | 1.1 | 0.29 |
| GPC4 | 0.065 | 1.1 (0.95-1.2) | 1.1 | 0.29 |
| PLIN2 | 0.098 | 1.1 (0.92-1.3) | 1.1 | 0.29 |
| SLC6A6 | -0.11 | 0.89 (0.72-1.1) | 1.1 | 0.29 |
| PHKG1 | 0.12 | 1.1 (0.9-1.4) | 1.1 | 0.3 |
| PLAC8 | 0.085 | 1.1 (0.93-1.3) | 1.1 | 0.3 |
| B3GALT6 | -0.14 | 0.87 (0.67-1.1) | 1 | 0.31 |
| LDHC | -0.28 | 0.76 (0.44-1.3) | 0.99 | 0.32 |
| PGM1 | 0.15 | 1.2 (0.87-1.5) | 1 | 0.32 |
| PAM | 0.12 | 1.1 (0.88-1.5) | 0.94 | 0.33 |
| SLC37A4 | 0.18 | 1.2 (0.84-1.7) | 0.96 | 0.33 |
| SULT2B1 | -0.048 | 0.95 (0.87-1) | 0.95 | 0.33 |
| HK2 | 0.079 | 1.1 (0.92-1.3) | 0.9 | 0.34 |
| NR3C1 | 0.14 | 1.2 (0.86-1.5) | 0.9 | 0.34 |
| KIF5A | 0.29 | 1.3 (0.73-2.4) | 0.86 | 0.35 |
| SERPINE1 | 0.052 | 1.1 (0.94-1.2) | 0.87 | 0.35 |
| CP | 0.076 | 1.1 (0.91-1.3) | 0.81 | 0.37 |
| JUN | 0.089 | 1.1 (0.9-1.3) | 0.82 | 0.37 |
| PFKFB3 | 0.099 | 1.1 (0.89-1.4) | 0.82 | 0.37 |
| SLC2A5 | 0.099 | 1.1 (0.89-1.4) | 0.79 | 0.38 |
| BRS3 | -6.2 | 0.0021 (1e-09-4300) | 0.69 | 0.4 |
| KLF7 | 0.097 | 1.1 (0.88-1.4) | 0.7 | 0.4 |
| NDST2 | -0.59 | 0.55 (0.14-2.2) | 0.71 | 0.4 |
| TKTL1 | -0.085 | 0.92 (0.75-1.1) | 0.71 | 0.4 |
| ZFP36 | 0.068 | 1.1 (0.91-1.3) | 0.7 | 0.4 |
| BNIP3L | 0.1 | 1.1 (0.86-1.4) | 0.64 | 0.42 |
| ANKZF1 | 0.13 | 1.1 (0.82-1.6) | 0.61 | 0.43 |
| TMEM45A | -0.049 | 0.95 (0.84-1.1) | 0.63 | 0.43 |
| ALDOB | 0.36 | 1.4 (0.57-3.6) | 0.59 | 0.44 |
| FOXO3 | 0.12 | 1.1 (0.84-1.5) | 0.59 | 0.44 |
| PPP1R3C | 0.05 | 1.1 (0.92-1.2) | 0.56 | 0.45 |
| TPST2 | 0.14 | 1.1 (0.8-1.6) | 0.58 | 0.45 |
| DUSP1 | -0.053 | 0.95 (0.82-1.1) | 0.54 | 0.46 |
| BCAN | 0.16 | 1.2 (0.76-1.8) | 0.52 | 0.47 |
| DCN | -0.044 | 0.96 (0.85-1.1) | 0.53 | 0.47 |
| RORA | -0.12 | 0.89 (0.64-1.2) | 0.52 | 0.47 |
| KLF6 | -0.077 | 0.93 (0.74-1.2) | 0.46 | 0.5 |
| BCL2 | -0.11 | 0.9 (0.65-1.2) | 0.43 | 0.51 |
| PPFIA4 | 0.14 | 1.1 (0.76-1.7) | 0.43 | 0.51 |
| GAA | 0.062 | 1.1 (0.88-1.3) | 0.41 | 0.52 |
| IL6 | 0.038 | 1 (0.92-1.2) | 0.41 | 0.52 |
| CCNG2 | -0.084 | 0.92 (0.71-1.2) | 0.4 | 0.53 |
| ANGPTL4 | -0.043 | 0.96 (0.83-1.1) | 0.36 | 0.55 |
| PPARGC1A | 0.11 | 1.1 (0.78-1.6) | 0.35 | 0.55 |
| DPYSL4 | 0.067 | 1.1 (0.85-1.3) | 0.33 | 0.56 |
| BGN | 0.036 | 1 (0.92-1.2) | 0.33 | 0.57 |
| COL5A1 | -0.033 | 0.97 (0.86-1.1) | 0.32 | 0.57 |
| LALBA | -2.4 | 0.09 (2.1e-05-400) | 0.32 | 0.57 |
| MT2A | 0.035 | 1 (0.92-1.2) | 0.33 | 0.57 |
| RRAGD | 0.059 | 1.1 (0.87-1.3) | 0.33 | 0.57 |
| ZNF292 | 0.099 | 1.1 (0.77-1.6) | 0.3 | 0.58 |
| IGFBP3 | 0.032 | 1 (0.92-1.2) | 0.29 | 0.59 |
| PKP1 | -0.037 | 0.96 (0.84-1.1) | 0.28 | 0.59 |
| GLRX | -0.072 | 0.93 (0.71-1.2) | 0.27 | 0.6 |
| SDC3 | -0.066 | 0.94 (0.73-1.2) | 0.28 | 0.6 |
| FBP1 | 0.03 | 1 (0.92-1.2) | 0.26 | 0.61 |
| HAS1 | 0.052 | 1.1 (0.86-1.3) | 0.26 | 0.61 |
| PCK1 | 0.2 | 1.2 (0.55-2.7) | 0.24 | 0.62 |
| CAV1 | 0.032 | 1 (0.9-1.2) | 0.21 | 0.64 |
| PDK3 | 0.083 | 1.1 (0.77-1.5) | 0.22 | 0.64 |
| CXCR4 | -0.031 | 0.97 (0.84-1.1) | 0.19 | 0.66 |
| PIM1 | -0.047 | 0.95 (0.76-1.2) | 0.17 | 0.68 |
| CITED2 | -0.041 | 0.96 (0.78-1.2) | 0.16 | 0.69 |
| GAPDHS | 0.86 | 2.4 (0.029-190) | 0.15 | 0.7 |
| GALK1 | -0.055 | 0.95 (0.71-1.3) | 0.14 | 0.71 |
| EFNA3 | -0.032 | 0.97 (0.81-1.2) | 0.12 | 0.73 |
| INHA | 0.094 | 1.1 (0.65-1.9) | 0.12 | 0.73 |
| HDLBP | 0.06 | 1.1 (0.75-1.5) | 0.11 | 0.74 |
| LXN | 0.03 | 1 (0.86-1.2) | 0.11 | 0.74 |
| BTG1 | -0.037 | 0.96 (0.76-1.2) | 0.09 | 0.76 |
| PRDX5 | -0.039 | 0.96 (0.75-1.2) | 0.09 | 0.76 |
| MAP3K1 | -0.041 | 0.96 (0.73-1.3) | 0.08 | 0.77 |
| PGM2 | 0.032 | 1 (0.83-1.3) | 0.08 | 0.78 |
| MT1E | -0.014 | 0.99 (0.89-1.1) | 0.07 | 0.79 |
| VLDLR | -0.026 | 0.97 (0.8-1.2) | 0.06 | 0.8 |
| ETS1 | -0.022 | 0.98 (0.82-1.2) | 0.06 | 0.81 |
| SELENBP1 | -0.024 | 0.98 (0.81-1.2) | 0.06 | 0.81 |
| UGP2 | 0.051 | 1.1 (0.69-1.6) | 0.06 | 0.81 |
| PGF | 0.015 | 1 (0.86-1.2) | 0.04 | 0.85 |
| ILVBL | 0.023 | 1 (0.75-1.4) | 0.02 | 0.88 |
| PDGFB | 0.015 | 1 (0.83-1.2) | 0.02 | 0.88 |
| KDELR3 | -0.011 | 0.99 (0.84-1.2) | 0.02 | 0.89 |
| MYH9 | -0.017 | 0.98 (0.77-1.2) | 0.02 | 0.89 |
| PLAUR | 0.014 | 1 (0.82-1.3) | 0.02 | 0.89 |
| CHST3 | 0.016 | 1 (0.8-1.3) | 0.02 | 0.9 |
| HMOX1 | -0.011 | 0.99 (0.83-1.2) | 0.02 | 0.9 |
| TGFBI | 0.0074 | 1 (0.9-1.1) | 0.02 | 0.9 |
| CDKN1C | 0.01 | 1 (0.85-1.2) | 0.01 | 0.91 |
| PNRC1 | 0.012 | 1 (0.77-1.3) | 0.01 | 0.93 |
| LOX | -0.0051 | 0.99 (0.87-1.1) | 0.01 | 0.94 |
| RBPJ | -0.0095 | 0.99 (0.72-1.4) | 0 | 0.95 |
| GCK | -0.0064 | 0.99 (0.63-1.6) | 0 | 0.98 |
| IGFBP1 | -0.011 | 0.99 (0.38-2.6) | 0 | 0.98 |
| CDKN1A | 0.0012 | 1 (0.78-1.3) | 0 | 0.99 |
| FOSL2 | -0.0017 | 1 (0.76-1.3) | 0 | 0.99 |
| SDC2 | 0.00028 | 1 (0.87-1.1) | 0 | 1 |

Table S2: Regression coefficients of 5 prognostic hypoxia genes.

| Genes | Coefficient |
| --- | --- |
| CNFN | -0.282931693 |
| AQP3 | 0.094246012 |
| KRT10 | 0.20580369 |
| KRT8 | 0.190962707 |
| DDIT4 | 0.245061427 |
